# Nanomechanics of the Endothelial Glycocalyx in Experimental Sepsis

**DOI:** 10.1371/journal.pone.0080905

**Published:** 2013-11-20

**Authors:** Anne Wiesinger, Wladimir Peters, Daniel Chappell, Dominik Kentrup, Stefan Reuter, Hermann Pavenstädt, Hans Oberleithner, Philipp Kümpers

**Affiliations:** 1 Department of Medicine D, Division of General Internal Medicine, Nephrology, and Rheumatology, University Hospital, Muenster, Muenster, Germany; 2 Institute of Physiology II, University of Muenster, Muenster, Germany; 3 Clinic of Anesthesiology, Ludwig-Maximilians-University Munich, Munich, Germany; Tufts University, United States of America

## Abstract

The endothelial glycocalyx (eGC), a carbohydrate-rich layer lining the luminal side of the endothelium, regulates vascular adhesiveness and permeability. Although central to the pathophysiology of vascular barrier dysfunction in sepsis, glycocalyx damage has been generally understudied, in part because of the aberrancy of *in vitro* preparations and its degradation during tissue handling. The aim of this study was to analyze inflammation-induced damage of the eGC on living endothelial cells by atomic-force microscopy (AFM) nanoindentation technique. AFM revealed the existence of a mature eGC on the luminal endothelial surface of freshly isolated rodent aorta preparations *ex vivo*, as well as on cultured human pulmonary microvascular endothelial cells (HPMEC) *in vitro*. AFM detected a marked reduction in glycocalyx thickness (266 ± 12 vs. 137 ± 17 nm, P<0.0001) and stiffness (0.34 ± 0.03 vs. 0.21 ± 0.01 pN/mn, P<0.0001) in septic mice (1 mg *E. coli* lipopolysaccharides (LPS)/kg BW i.p.) compared to controls. Corresponding *in vitro* experiments revealed that sepsis-associated mediators, such as thrombin, LPS or Tumor Necrosis Factor-α alone were sufficient to rapidly decrease eGC thickness (-50%, all P<0.0001) and stiffness (-20% P<0.0001) on HPMEC. In summary, AFM nanoindentation is a promising novel approach to uncover mechanisms involved in deterioration and refurbishment of the eGC in sepsis.

## Introduction

Endothelial hyperpermeability is a hallmark of systemic inflammatory response syndrome (SIRS) and sepsis that largely contributes to high morbidity and mortality in critically-ill patients. The often devastating clinical consequences of this process are net extravasation of fluid, a profound decrease in systemic vascular tone, and collapse of the microcirculation, leading to distributive shock, acute lung and kidney injury [1-3]. Inflammation-induced vascular leakage has long been ascribed to a malfunction of the endothelial cell itself. However, recent studies provided compelling evidence that the endothelium is protected against pathogenic insults by a highly hydrated negatively charged “firewall” on the luminal side called the *glycocalyx* [4-6]. Given its strategic location as the interface between the blood and the endothelium, the intact glycocalyx mediates flow-induced shear stress on endothelial cells, prevents transvascular protein leakage and reduces leukocyte-endothelial interactions [5,7,8].

The endothelial glycocalyx (eGC) is a carbohydrate-rich gel-like mesh of large anionic polymers covering the luminal surface of endothelium along the entire vascular tree. The most prominent components of the eGC are the proteoglycans, especially those of the syndecan family, to which both highly sulfated glycosaminoglycans (mainly heparan- and chondroitin sulfates) and hyaluronan are attached [9,10]. Along with various incorporated proteins of plasmatic and endothelial origin, the eGC attains thickness of up to 2 µm and thus is considerably thicker than the endothelial cells themselves [9,11-15]. Under physiological conditions, the structure of the glycocalyx layer is fairly stable but subject to a permanent dynamic balance between biosynthesis of new glycosaminoglycans and shear dependent removal of existing constituents.

Selective thinning of the eGC by enzyme digestion promotes microvascular hyperpermeability [16-19] and exposes previously hidden endothelial surface adhesion molecules including ICAM-1 and VCAM-1, allowing neutrophil recognition of, and adhesion to, the endothelial surface [5,20]. It has been shown that loss of the eGC constituents - and as a consequence softening of the eGC - can lead to changes in microvascular rheology and haemodynamics [21,22]. Observational studies in critically ill patients with sepsis indicate that plasma levels of shed glycocalyx constituents correlate with disease severity and mortality [23-25]. 

However, despite its fundamental role in regulating vascular integrity and functions central to the pathophysiology of sepsis, there is a lack of methods to visualize and quantify glycocalyx deterioration *in vitro*. The atomic force microscope (AFM) fascilitates quantitative analysis of nanomechanical properties of living cells [26-28]. In a recent proof-of-principle study, we successfully employed AFM nanoindentation to disclose the eGC on split-open arteries from a human umbilical cord [29]. Here, we aimed to employ this novel methodology to analyze sepsis-induced (nano-) mechanical changes of the eGC *ex vivo* and *in vitro* for the first time.

## Materials and Methods

### *In vivo* animal studies

All procedures were approved by a governmental committee on animal welfare (Landesamt für Natur, Umwelt und Verbraucherschutz Nordrhein-Westfalen), performed according to international animal protection guidelines and all efforts were made to minimize suffering. Eight to fourteen week-old male Lewis-Brown Norway rats (weighing 250–380 g) and eight-week-old male C57BL/6 mice (weighing 20–25 g) were obtained from Janvier (Janvier, Le Genest Saint Isle, France) and Charles River (The Charles River Laboratories; Sulzfeld, Germany). All animals had free access to standard chow and tap water, and were acclimated to the facility for at least one week before beginning an experiment.

#### Enzymatic degradation of the endothelial glycocalyx in rats

Rats were anesthetized with ketamine 100 mg/kg body weight (BW) intraperitoneal (i.p.) and xylazine 5 mg/kg BW i.p. (CEVA Tiergesundheit, Düsseldorf, Germany). Enzymatic degradation of the endothelial glycocalyx *in vivo* was induced by infusing heparinase I (140 Sigma-Units/kg BW; Sigma, St. Louis, MO, USA) via the tail vein. An equal amount of solvent (0.9% NaCl) served as control. Aortas (n = 5 per group) were harvested 45 min after injection of heparinase or solvent.

#### Endotoxemia in mice

Mice were intraperitoneally injected with 1 mg/kg BW lipopolysaccharide (LPS) from *Escherichia coli*, serotype O111:B4 (Sigma-Aldrich, St. Louis, MO). An equal amount of solvent (phosphate-buffered saline - PBS) served as control. Aortas (n = 4 per group) were harvested 18 h after administration of LPS or solvent.

#### Tissue harvest and preparation

**Figure 1:**
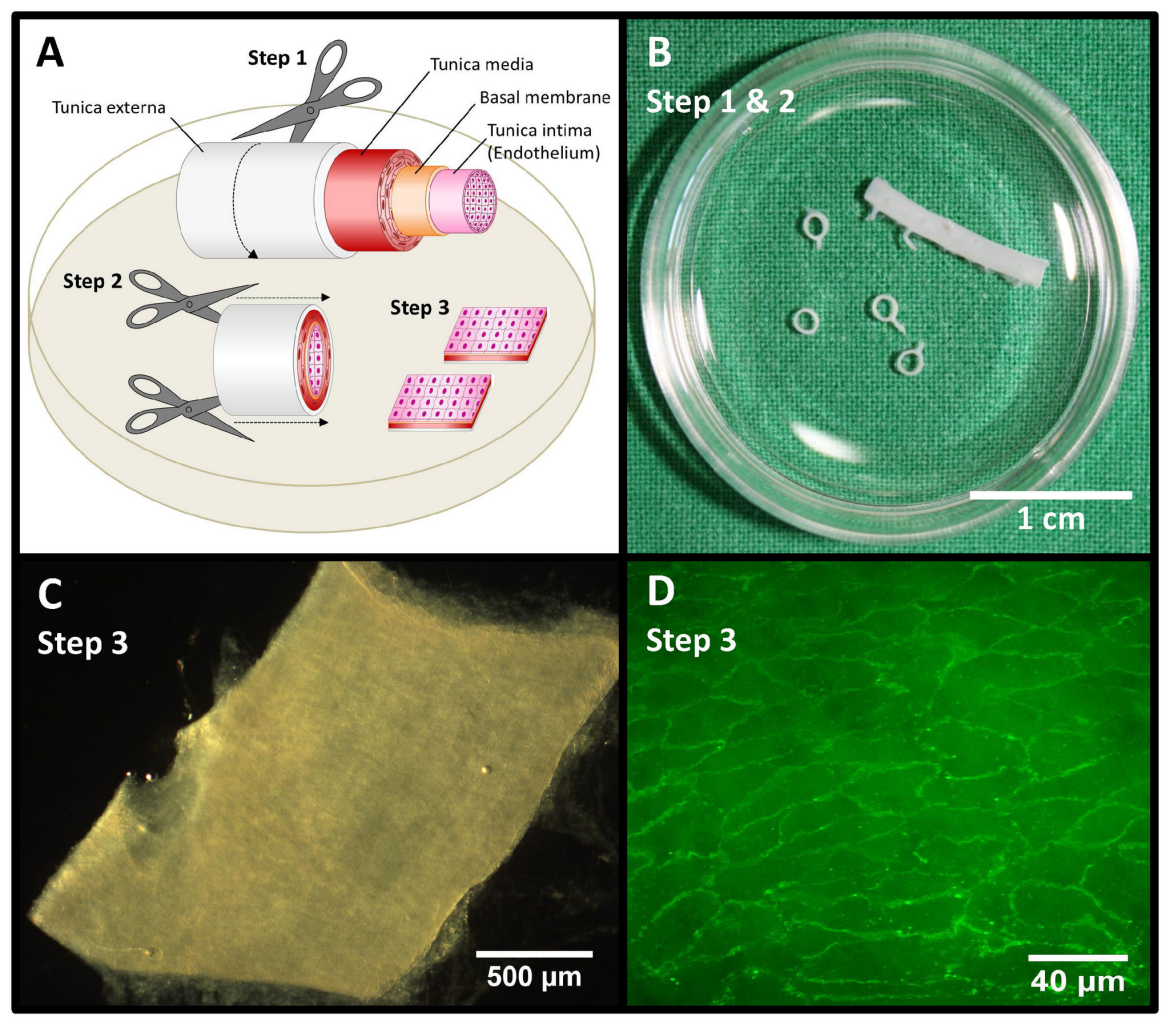
Preparation of rat aorta for AFM analysis *ex* *vivo*. (A-C) Aorta was isolated, freed from surrounding tissue and cut in rings. The aortic rings were split into small preparations (approximately 4 mm^2^). For atomic force microscopy (AFM) analysis aorta preparations were attached on glass coverslips with the endothelial surface facing upwards. (D) Preservation of the endothelial cell layer after preparation was approved by immunofluorescence staining for CD31 (PECAM-1).

Harvest and preparation of aortas for *ex vivo* analysis by AFM is visualized in Figure **1**. Aortas were perfused with PBS (PAA Laboratories, Pasching, Austria) with 1% Penicillin and Streptomycin (Biochrom AG, Berlin, Germany) via cardiac puncture, isolated, immediately bathed in PBS with 1% Penicillin/Streptomycin (Pen/Strep) and freed from surrounding tissue. Small patches (~4 mm^2^) of the whole aorta were attached on Cell-Tak® (BD Biosciences, Bedford, MA, USA) coated glass, with the endothelial surface facing upwards. After preparation, the patches were bathed in minimal essential medium (MEM; Invitrogen Corp., La Jolla, CA, USA) supplemented with 20% fetal calf serum (FCS; PAA Laboratories, Pasching, Austria), 1% MEM vitamins (Invitrogen), 1% MEM nonessential amino acids (Invitrogen) and 1% Pen/Strep.

### Experimental conditions

Indentation measurements on aorta preparations or cultured endothelial cells (see below) were performed before and after enzymatic degradation by various doses (see below) of heparinase I, hyaluronidase, or chondroitinase (all Sigma) for 60 min at 37°C unless stated otherwise. In addition, indentation measurements on cultured endothelial cells were performed before and after challenge with the following sepsis mediators for 60 min at 37°C: a) thrombin (1 U/ml, Sigma), b) *E. coli* LPS (100 ng/ml, Sigma) plus LPS-binding protein (100 ng/ml, Sigma) plus CD14 (10 ng/ml, Sigma) or c) Tumor Necrosis Factor (TNF)-α (10 ng/ml, Peprotech, Hamburg, Germany) dissolved in sterile water (Braun AG, Melsungen, Germany).

### Immunofluorescence Microscopy

After preparation, aortic patches were fixed for 15 minutes in 4% paraformaldehyde (30% Formaldehyde, Carl ROTH GmbH, Karlsruhe, Germany) at 4°C. Following fixation, samples were permeabilized for 5 min in 0.1 % Triton X-100 (Carl ROTH GmbH, Karlsruhe, Germany) at 4°C, blocked with 10% normal goat serum (Sigma, St. Louis, MO, USA) for 30 minutes, and incubated for 1 hour with the primary antibody (polyclonal rabbit anti-human CD31 (PECAM) antibody, Abbiotec, San Diego, CA, USA). After serial washes in PBS, the samples were incubated with the Fluorophore-conjugated secondary antibody (goat anti-rabbit Alexa Fluoro 488, Invitrogen Molecular Probes, Eugene, Oregon, USA) for 60 minutes. Coverslips were mounted with Dako Fluorescent Mounting Medium (Dako, Via Real, Carpinteria, USA). Images were taken with a ZEISS Axio Observer (Zeiss, Jena, Germany) using the Meta Morph software version 7.5.6.0 (Molecular Devices, Biberach an der Risse, Germany).

### Electron Microscopy

For electron microscopy rats were anaesthetized and perfused with a solution composed of 2% glutaraldehyde, 2% sucrose, 0.1 M sodium cacodylate buffer (pH 7.3), and 2% lanthanum nitrate through a cannula placed in the left ventricle. Thereafter, the aorta was harvested diced. Three to four pieces of approximately 1 mm^3^ each were immersed in the fixation solution for 2 h and remained overnight in a solution without glutaraldehyde before being washed in alkaline (0.03 mol/l NaOH) saccharose (2%) solution. After contrast enhancement with a solution containing 2% osmium tetroxide and 2% lanthanum nitrate, embedding in araldite, and microtomic sectioning, electron microscopy of the eGC was performed as previously described [4].

### Atomic force microscopy

**Figure 2:**
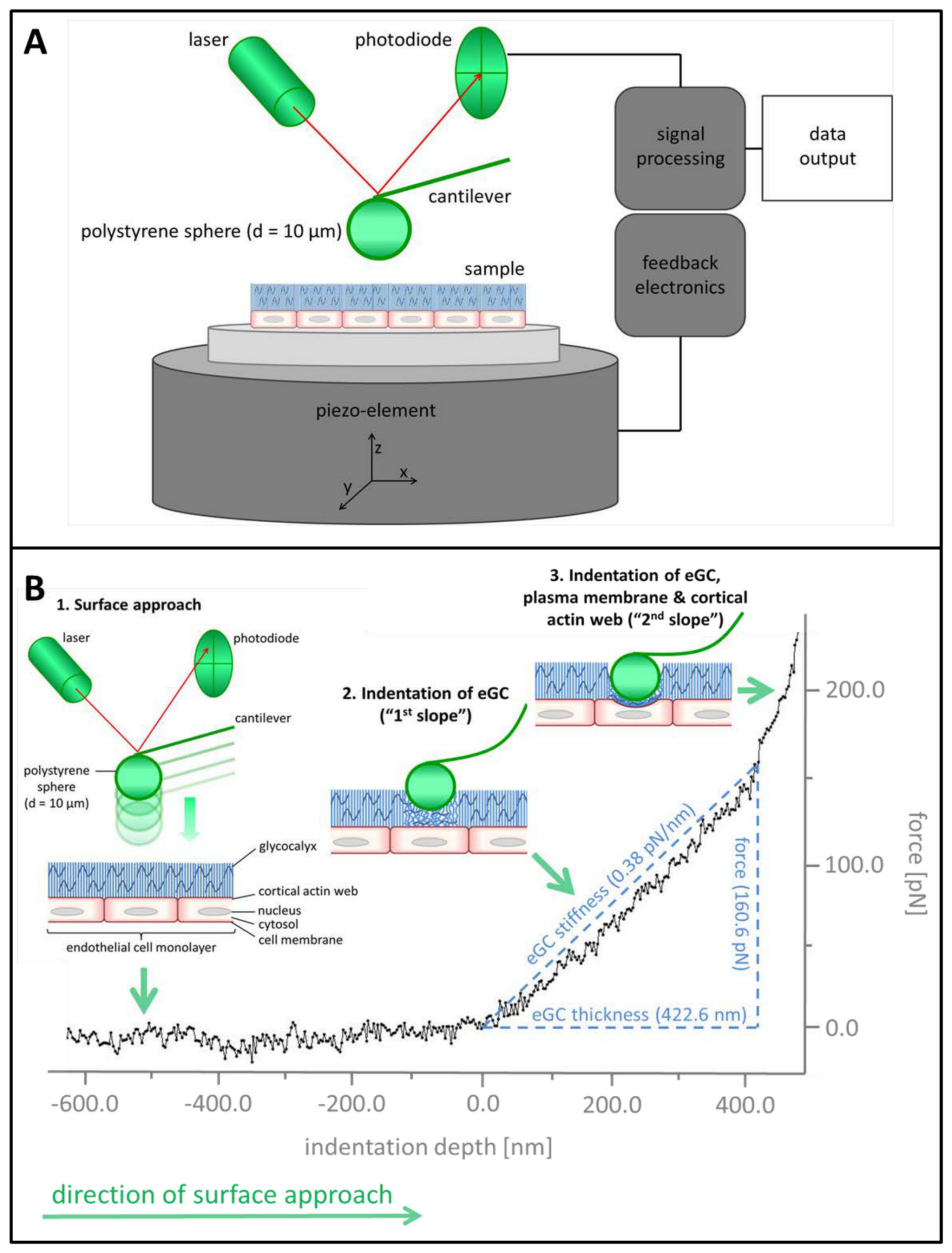
Setup of the atomic force microscope (AFM) used in this study (A) for nanomechanical analysis of the endothelial glycocalyx (eGC) and an original tracing of a force indentation curve performed on the endothelial surface of a rat aorta (B) *ex vivo.* 1) The AFM tip travels vertically towards the endothelial surface. 2) Upon indentation of the endothelial glycocalyx (eGC), the AFM cantilever, which serves as a soft spring, is deflected. The cantilever deflection is measured as a laser beam reflected from the back of the cantilever and is plotted as a function of sample position along the z axis. The resulting curve is transformed into a force-versus-indentation curve using the cantilever’s spring constant and the optical lever sensitivity. The slope of a force indentation curve then directly reflects the stiffness (expressed in pN/nm), which is necessary to indent the eGC for a certain distance. The first slope indicates the stiffness (in this trace 0.38 pN/nm) of the very first layer, the endothelial glycocalyx (eGC). The second slope indicates the stiffness of the plasma membrane and the cortical actin web. Practically, the slope of a force indentation curve is deduced from a manually plotted regression line (similar to the line in the trace) in PUNIAS (Protein Unfolding and Nano-Indentation Analysis Software, Version 1.0, Release 1.8, http:// punias.voila.net/). In our experience, this slope is virtually linear over its entire length, resulting in a single fixed stiffness value. The distance between the starting point of eGC indentation and the starting point of the second slope (projected to the x-axis) corresponds to the thickness of the eGC (in this trace 422.6 nm).

Thickness and stiffness of the eGC were determined using the Atomic Force Microscope (AFM) nanoindentation technique. Preservation of the endothelial cell layer on aorta preparations was approved by immunostaining of PECAM-1/CD31 (Figure **1**). Figure **2 A, B** illustrates the basic principles of this method. By using a Multimode AFM (Veeco, Mannheim, Germany) with a feedback-controlled heating device (Veeco) measurements were performed at 37°C as described previously [29]. 

In brief, the central component of the AFM is a very sensitive mechanical nanosensor – a triangular cantilever with a mounted spherical tip (here: electrically uncharged polystyrene, diameter = 10 µm, Novascan, Ames, IA, USA) that is utilized to periodically indent the cells. A spherical tip was used for this AFM approach instead of a sharp tip because of a larger interaction area between tip and sample that decreases the effective pressure and results in less mechanical noise [30]. The cantilever functions as a soft spring (spring constant = 11 pN/nm). The *xyz*-position of the tip is precisely controlled by a piezo-element (Figure **2 A**). A laser beam is reflected by the gold-coated backside of the cantilever to a position-sensitive quadrupled photodiode allowing measurements of the cantilever deflection (*V*). Determination of the spring constant (*K*_*cant*_) by the thermal tuning method and measurement of the deflection sensitivity (*α*) of the cantilever on bare glass coverslips facilitate the calculation of the force (*F*) acting on the cantilever and, in turn, the force exerted by the cantilever to the sample. 

$$F=V\cdot \alpha \cdot K_{cant}$$

Since the piezo displacement (*x*_*piezo*_) and the deflection sensitivity (*α*) are known, the indentation depth (deformation) of the sample (*x*_*sample*_) can be calculated. 

$$x_{sample}=x_{piezo}-(\alpha \cdot V)$$

For reasons of readability the indentation depth is hereafter called “thickness”. It should be noted that the indentation depth rather represents an *apparent* thickness, rather than the exact *anatomical* thickness. 

Force indentation curves of a single cell were obtained by plotting the force (*F*) necessary to indent the cell (indentation depth, *x*_*sample*_). The sample stiffness can be derived from Hook´s law.

$$K_{sample}=\frac{F}{x_{sample}}$$

The stiffness (*K*) is the mechanical resistance of a sample against a defined deformation (e.g. indentation). *K* depends strongly on the indentation depth and the location, because cells contain a variety of substructures and organelles. The experimental parameters including an indentation velocity of 1 µm/s, a loading force of approximately 400 pN, an indentation frequency in the range of 0.25 - 0.5 Hz, a ramp size of 2 µm, a trig threshold of 35 nm and a tip velocity of 0.5 - 1 µm/s. 

Previous experiments, using 1 µm AFM-tips, showed that the glycocalyx thickness is somewhat variable [29,31]. Since we were interested in the overall condition of the glycocalyx and especially in its changes induced by different stimuli, we here chose larger tips (10 µm), as they indent a larger area. Thus they provided “more averaged” results and enabled us to avoid the data being influenced by the spatial distribution of the eGC thickness. All measurements were performed in HEPES-buffered solution [standard composition in millimolars: 140 NaCl, 5 KCl, 1 MgCl_2_, 1 CaCl_2_, 5 Glucose, 10 HEPES (N-2-hydroxyethylpiperazine-N′-2-ethanesulfonic acid), pH 7.4] supplemented with 1% FCS in order to prevent eGC collapse [32]. 

Light microscopy was used to ensure that the tip position of the mechanical nanosensor was located neither at the nuclear, nor at the junctional region of cultured endothelial cells. However, this approach was not feasible in (thick) explanted aortas due to the lack of transparency of sub-endothelial layers such as the *Tunica media* and *T. externa*.

**Figure 3:**
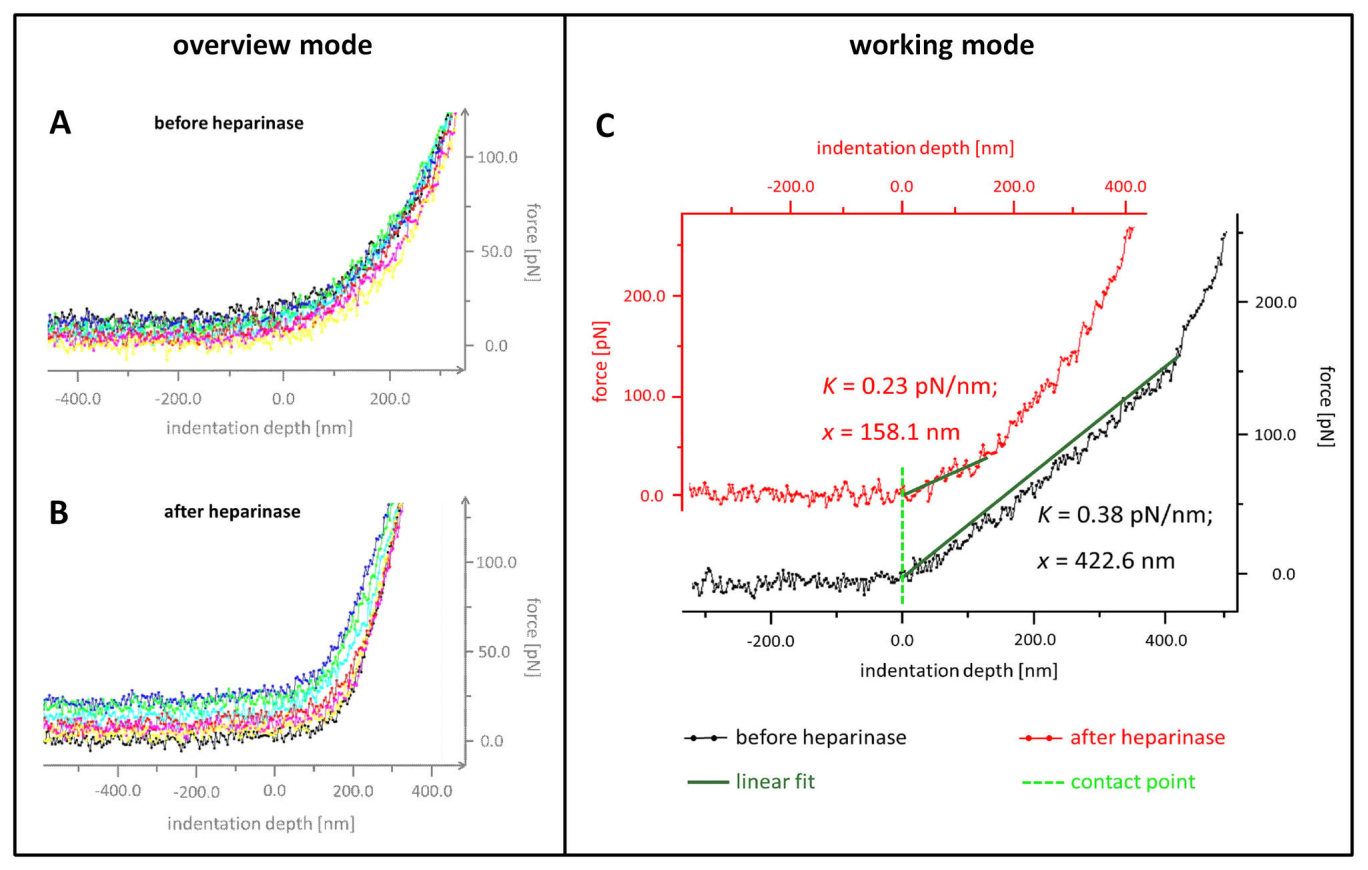
Original force indentation tracings before (A, C) and after (B, C) heparinase I treatment measured on *ex* *vivo* rat aortic samples by AFM. (A, B) “Overview mode” showing 7 force indentation curves recorded from a single endothelial cell before and after heparinase I treatment, respectively. (C) Each force indentation curve was then analyzed separately using a higher magnification (“working mode”) which facilitates the determination of the thickness and the stiffness of the endothelial glycocalyx by linear approximation. Please note that values on X- and Y-axis in A and B are approximations for graphical illustration.

Figure **3 A, B** show typical force indentation curves of an untreated as well as heparinase-treated aortic endothelial cell (“overview mode”). Each force indentation curve was then analyzed separately with a higher magnification (“working mode”) by using a linear approximation for determination of the eGC nanomechanics (Figure **3 C**).

### Cell culture

*Primary human umbilical venous endothelial cells* (*HUVECs*) were isolated and grown in culture as previously described [33]. Cells were maintained in M199 (Gibco, Karlsruhe, Germany) supplemented with 10% heat-inactivated fetal calf serum (FCS; PAA Laboratories, Pasching, Austria), 1% Pen/Strep, 50 U/mL heparin (Biochrom AG) and 1% growth supplement derived from bovine retina. Confluent HUVECs of the first or second passage were used for experiments. The *human umbilical vein endothelial cell line EA.hy 926* [34] was grown in Dulbecco's modified Eagle's medium (DMEM; Invitrogen, Karlsruhe, Germany) supplemented with 1% Pen/Strep, and 10% FCS. The *human pulmonary microvascular endothelial cell line HPMEC-ST1.6R* [35] was grown in M199 medium (Gibco, Karlsruhe, Germany), supplemented with 20% FCS, glutamax at 2 mM (Invitrogen, Darmstadt, Germany), 1% Pen/Strep, sodium heparin at 25 mg/ml, and endothelial growth factor supplement at 25 μg/ml (BD Biosciences, Bedford, MA, USA). *The bovine aortic endothelial cell line GM 7373* (DSMZ, Braunschweig, Germany) was grown in minimal essential medium (MEM; Invitrogen) supplemented with 20% FCS, 1% MEM vitamins, 1% MEM nonessential amino acids and 1% Pen/Strep as described [29]. The *murine brain microvascular endothelial cell line bEnd.5* [36] was cultured in DMEM supplemented with 10% FCS, 1% L-glutamine (Biochrom AG, Berlin, Germany), 1% non-essential amino acids, 100 mM sodium-pyruvate (Fisher Scientific), 25 mM β-Mercaptoenthanol (Sigma, St. Loius, MO, USA) and 1% Pen/Strep. All cells were cultivated in T25 culture flasks. For AFM analysis, cells were grown for 6 days on glass coverslips [bEnd.5 cells were cultured on glass coverslips coated with 0.5% gelatine (Sigma–Aldrich Chemie GmbH, Steinheim, Germany), EA.hy926 and HPMEC-ST1.6R were grown on fibronectin-coated glass coverslips (Roche Diagnostics GmbH, Mannheim, Germany)] at 37°C and 5% CO_2_.

### Statistical analysis

Data are presented as absolute value with mean, or mean with standard error of the mean (SEM). Differences between two groups were analyzed by Student’s t-test. Differences between ≥ 3 groups were analyzed by using the one-way analysis of variance (ANOVA) with Bonferroni’s multiple comparison test. All tests were two-sided and significance was accepted at P<0.05. GraphPad Prism Version 5.02 (GraphPad Prism Software Inc, San Diego, California, USA) was used for data analysis and figure preparation.

## Results

### Nanomechanical changes of the eGC after enzymatic degradation

**Figure 4:**
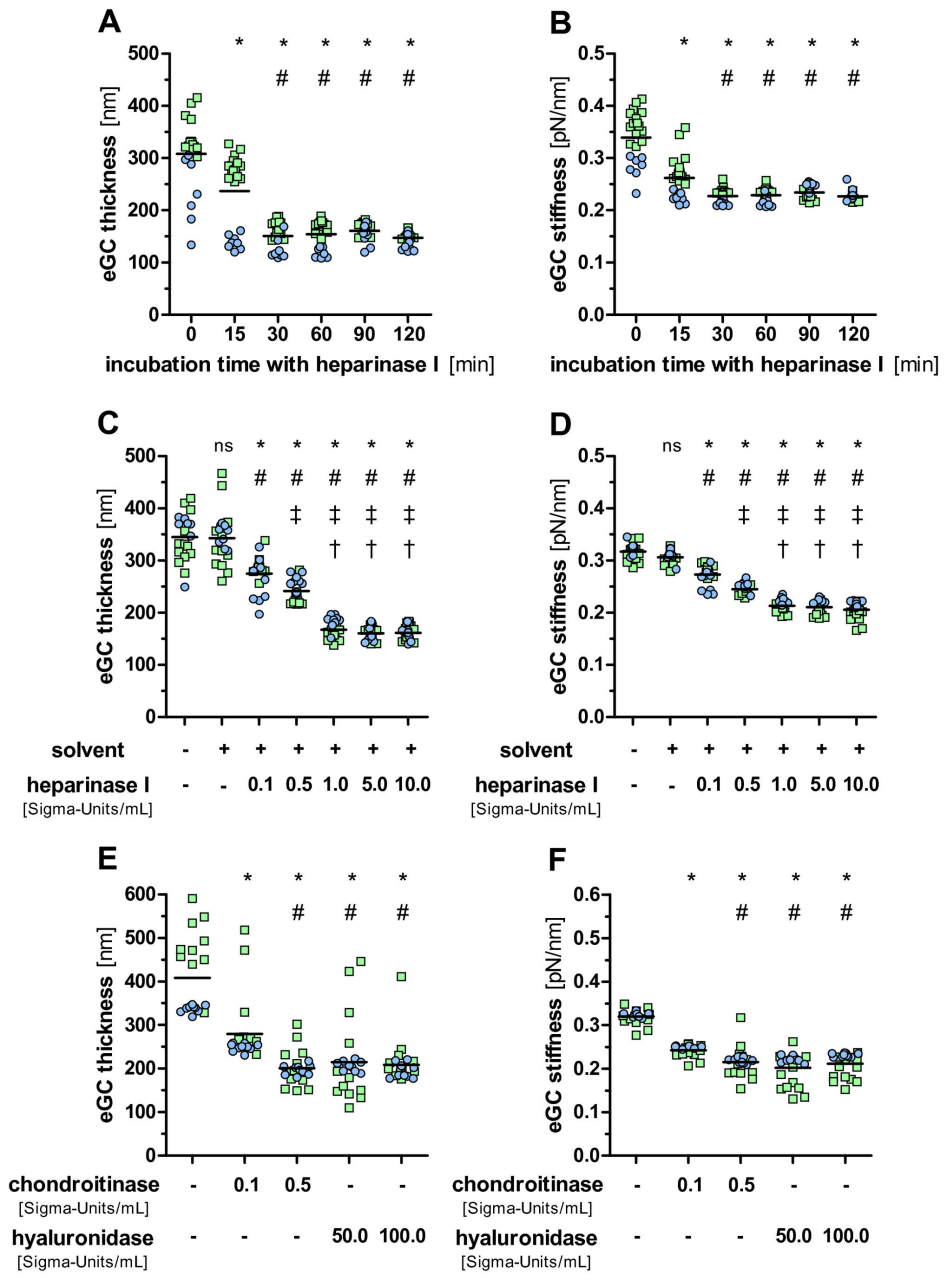
Time- and dose-dependent effects of enzyme digestion on eGC thickness and stiffness. Enzymatic removal of heparan sulfate residues by heparinase I *in* *vitro* led to a time- and dose-dependent reduction of eGC thickness (A, C) and stiffness (B, D) on freshly-isolated rat aorta preparations. Similar results were obtained using hyaluronidase and chondroitinase (E, F) to strip the eGC of hyaluronan and chondroitin sulfate, respectively. Data shown are from independent AFM experiments using paired aorta preparations derived from two rat aortas (indicated by either green or blue dots). Each dot represents an average of ≥5 force indentation curves per tip-position (i.e. per individual endothelial cell). *P<0.0001 versus untreated; #P<0.01 vs. 15 min, or solvent, or 0.1 S-U/mL chondroitinase, respectively; ‡*P*<0.001 vs. 0.1 Sigma Units/mL heparinase I; †*P*<0.0001 vs. 0.5 Sigma Units/mL heparinase I; ns, not significant. Horizontal bars indicate mean values.

A reductionist approach used by many groups, including ours, is to elucidate the roles and functions of different eGC constituents using enzyme digestions. Initially, we used freshly-isolated aortic preparations from untreated rats to mimic *physiological* conditions as closely as possible. Similar to previous investigations [28,31,37,38] two different slopes could be identified depending on the indentation depth of the cantilever. The first slope (indentation depth of the first hundred nanometers) represents the stiffness of the eGC, whereas the second steep slope reflects the stiffness of the plasma membrane with the underlying cortical actin web (cell cortex) (Figure **2 B**). Time-course and dose-response experiments showed that within 60 minutes after addition of heparinase I (1 Sigma-Unit/mL), which cleaves heparan sulfate residues, glycocalyx thickness and stiffness decreased by a maximum of approximately 50% (from 308 ± 68 nm to 154 ± 26 nm, *P*<0.0001) and 33% (from 0.34 ± 0.05 pN/nm to 0.23 ± 0.01 pN/nm, *P*<0.0001), respectively (Figure **4 A, B**). Neither longer exposure times, nor higher doses of heparinase could reduce thickness and stiffness further (Figure **4 A-D**). Of note, digestion of the eGC with heparinase I had no obvious effect on the following part (cell cortex) of the force separation curve (Figure **S1**). Addition of heparin, a natural substrate for heparinase, completely abolished these changes, excluding non-enzymatic effects of heparinase treatment (Figure **S2**). In addition, we used hyaluronidase and chondroitinase to strip the eGC of two other, highly abundant components, namely hyaluronan and chondroitin sulfate. Interestingly, nanomechanical changes were identical to those seen after heparinase I treatment, i.e. both, thickness and stiffness dropped by approximately 50% (428 ± 105 nm to 209 ± 48 nm for hyaluronidase and 201 ± 36 nm for chondroitinase, both *P*<0.0001) and 35% (0.32 ± 0.02 pN/nm to 0.21 ± 0.03 pN/nm for hyaluronidase and 0.22 ± 0.03 pN/nm for chondroitinase, *P*<0.0001) (Figure **4 E, F**). In line with previous *in vivo* data [39], the combination of heparinase, hyaluronidase and chondroitinase reduced thickness and stiffness even more effectively than the individual enzymes (Figure **S3**). All of the above mentioned results were highly reproducible. In summary, this data indicates that enzymatic removal of any eGC constituents alters its nanomechanics in a relatively rapid and uniform fashion.

### Proof of principle that the endothelial surface layer detected by AFM is the eGC

**Figure 5:**
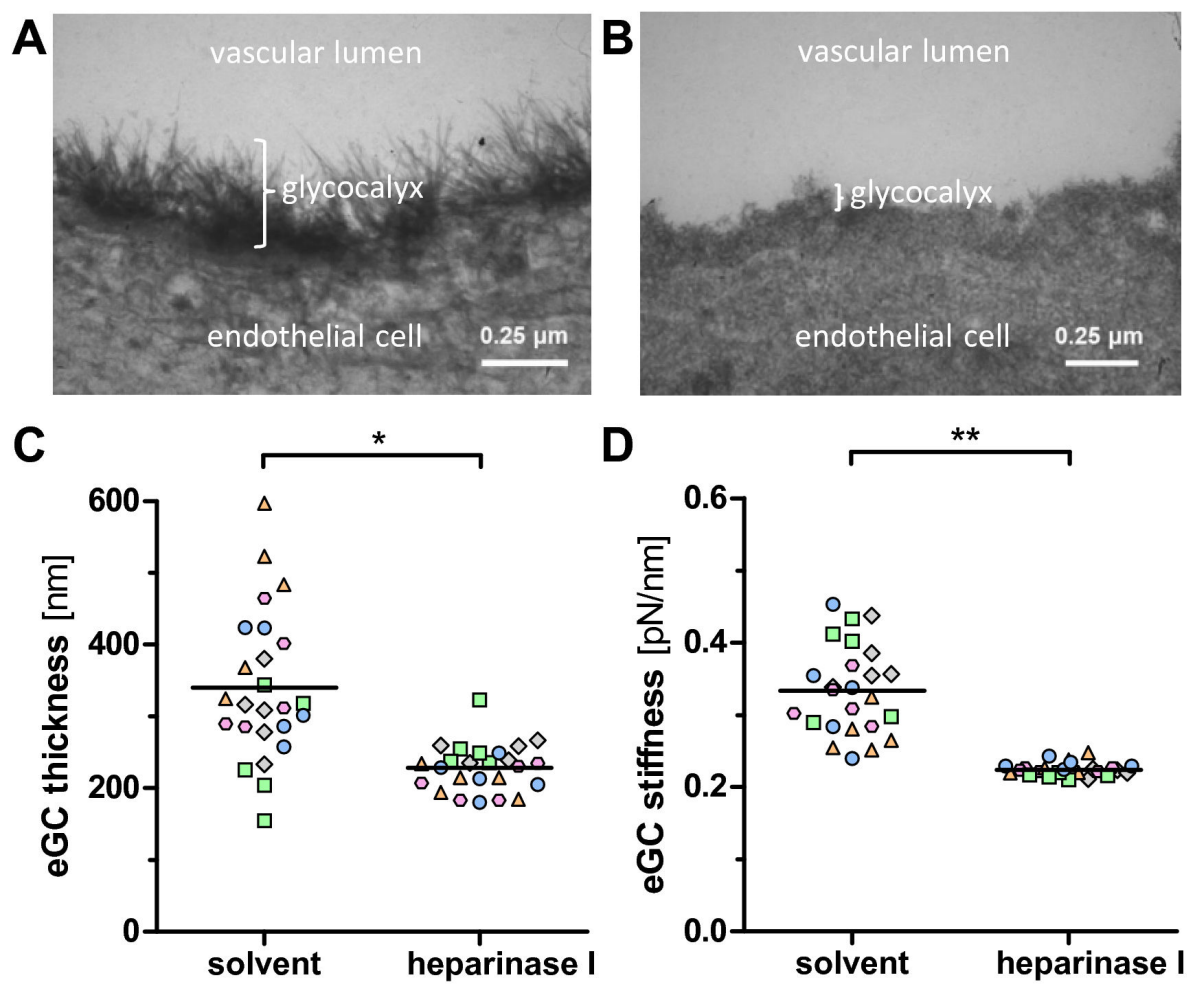
Proof of principle that the endothelial surface layer detected by AFM is the eGC. Enzymatic degradation of the eGC *in* *vivo* was induced by infusing healthy rats with heparinase I or solvent (n=5 per group, indicated by different color/shape). After 45 min aortas were harvested for transmission electron microscopy (EM) or AFM analysis, respectively. The heparinase-induced loss of filaments and thinning of the aortic eGC observed by EM (A, B) corresponded well with the decrease in eGC thickness detected by AFM (C, D). Each dot refers to a different tip position showing an average value of ≥5 force indentation curves. For statistical comparison, a single average value per animal was calculated. *P<0.05, **P<0.001. Horizontal bars indicate mean values.

To show that the indentation measurements indeed are able to disclose the eGC, we performed AFM and electron microscopy in a paired animal study. To avoid any fixation-bias (poor results with immersion-perfusion *in vitro* or after lengthier interruption of blood flow [14]), aortas were perfusion-fixed *in situ* (the gold standard for imaging the eGC structure). Electron microscopy revealed a homogenous and well-preserved glycocalyx on aortic endothelium from solvent-treated rats. Forty-five minutes after injection of heparinase *in vivo*, thinning and loss of filaments of the endothelial glycocalyx as observed by electron microscopy was paralleled by a reduction in thickness and stiffness, respectively (Figure **5**). Taken together, the data provides further evidence, that the heparinase-sensitive endothelial surface layer, disclosed by AFM measurements, is indeed the eGC.

### Deterioration of the eGC during murine endotoxemia

**Figure 6:**
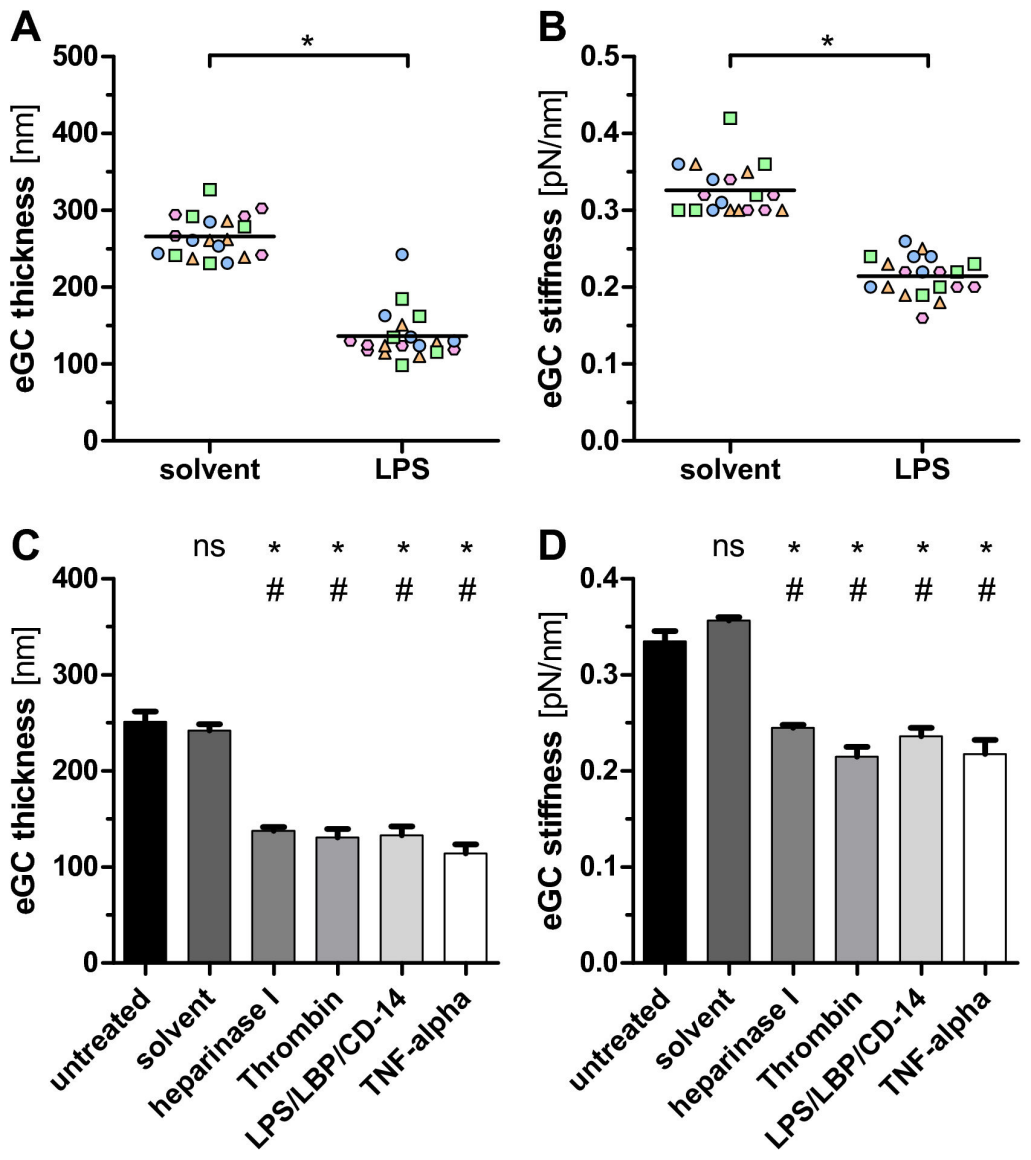
Damage of the eGC in sepsis. LPS challenge *in* *vivo* led to a decrease in eGC thickness (A) and stiffness (B). Adult mice were injected with Lipopoly-saccharides (LPS) from *E. coli* (1 mg/kg BW) or solvent (n=4 per group, indicated by different color/shape). After 18 h, aortas were harvested and immediately analyzed for eGC by AFM. Each dot refers to a different tip position showing an average value of ≥5 force indentation curves. For statistical comparison, a single average value per animal was calculated. *P<0.001. Treatment with either heparinase I, thrombin, LPS or TNF-α alone for 60 min was sufficient to decreased eGC thickness (C) and stiffness (D) on human pulmonary microvascular endothelial cells (HPMEC) *in* *vitro*. Data are expressed as mean (+/- standard error of mean) of n=3 samples per treatment condition. The average value per sample was calculated from a total of 25 force indentation curves, derived from five different tip-positions. *P<0.0001 versus untreated; ^#^P<0.0001 vs. solvent; ns, not significant.

Several lines of evidence suggest that endotoxin-induced inflammatory reactions lead to perturbation of the endothelial glycocalyx [40-42]. We therefore asked whether our AFM nanoindentation protocol can depict endothelial glycocalyx deterioration during murine endotoxemia. After having excluded differences in terms of thickness and stiffness between untreated mice and rats (Figure **S4**), adult mice were injected i.p. with a relatively low dose of LPS (1 mg/kg BW) or vehicle. After 18 h, aortas were harvested and immediately analyzed for endothelial glycocalyx by AFM. Indeed, AFM reliably detected a marked reduction in glycocalyx thickness (266 ± 17 vs. 137 ± 16 nm, *P*<0.0001) and stiffness (0.34 ± 0.03 vs. 0.21 ± 0.01 pN/mn, *P*<0.001) in LPS challenged mice compared to solvent treated controls (Figure **6 A, B**).

### Sepsis mediators reduce glycocalyx thickness *in vitro*

**Figure 7:**
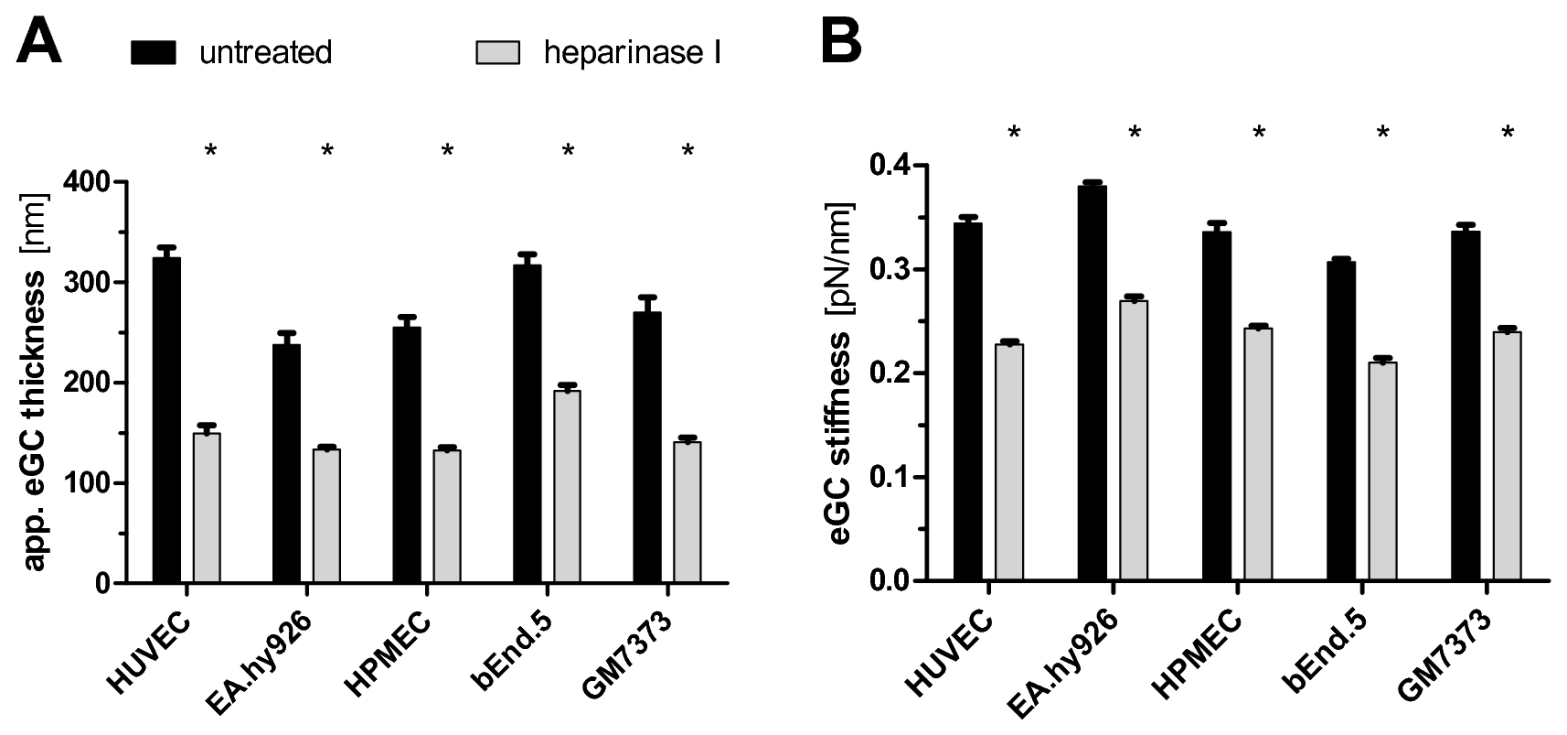
AFM measurements in various types of living endothelial cells *in* *vitro*. AFM nanoindentation measurements in various types of living endothelial cells *in* *vitro*, exposed to solvent or heparinase for 60 min. Enzymatic removal of heparan sulfate residues by heparinase I reduced eGC thickness (A) and stiffness (B) on confluent monolayers of human umbilical vein endothelial cells (HUVEC), immortalized human umbilical vein cells (EA.hy926), human pulmonal microvascular endothelial cells (HPMEC, clone ST1.6R), murine brain microvascular cells (bEnd.5), and bovine aortic endothelial cells (GM7373). Data are expressed as mean (+/- standard error of mean) of n=3 samples per treatment condition. The average value per sample was calculated from a total of ≥25 force indentation curves, derived from 15 different tip-positions. *P<0.0001.

Having observed this effect of LPS *in vivo*, we next tested whether LPS alone could reproduce glycocalyx deterioration *in vitro*. Using the identical AFM-protocol as with aorta preparations, we first ensured the existence of a fully-developed, heparinase-responsive eGC on human pulmonary microvascular endothelial cells (HPMEC) (Figure **6 C, D**) as well as on various other endothelial cell-lines and primary culture (Figure **7**). As expected, treatment with LPS alone decreased glycocalyx thickness and stiffness on HPMECs by 48% (*P*<0.0001) and 29% (*P*< 0.0001), respectively. Similarly, exposure to either thrombin or TNF-α alone led to a marked reduction of glycocalyx thickness by 48% / 55% (*P*<0.0001) and 36% / 35% (*P*<0.0001), respectively (Figure **6 C, D**). In summary, our data indicate that classical sepsis mediators, such as LPS, TNF-α, or thrombin alone are sufficient to rapidly deteriorate the eGC *in vitro*.

## Discussion

Whilst special electron microscopic staining procedures uncovered that the luminal surface of the endothelium expressed a carbohydrate layer by the mid-1940s, it was considered to be without major physiological relevance or functional significance for a long time. In the past two decades, however, intravital microscopy studies revealed that the eGC represents a substantial intravascular compartment contributing significantly to vascular wall homeostasis [7,43]. In the present study, we show that AFM can reliably depict nanomechanics of the eGC on a variety of living endothelial cells *ex vivo* and *in vitro*. Application of this technique in further experiments showed, that classical sepsis mediators, such as LPS, TNF-α, or thrombin alone are sufficient to rapidly deteriorate the eGC, as observed during murine endotoxemia.

Previous studies have reported a wide range of thickness values of the glycocalyx layer. This is partly due to different species and types of blood vessels used in experiments, and partly due to heterogeneous visualization methods employed. For example Gao and Lipowsky [39] detected an average eGC thickness of 463 nm in post-capillary venules of the intestinal mesentery of the rat using intravital microscopy to measure the exclusion zones for blood cells as opposed to fluorescent tracers. Berg et al. [19] could show by electron microscopy, that the rat myocardial capillary endothelial surface is coated with a 200 to 500 nm-thick carbohydrate layer. Using intravital fluorescent micro-particle image velocimetry in mouse cremaster muscle venules, Potter and Damiano detected a hydrodynamically relevant glycocalyx thickness of 520 nm [44]. In contrast to those impressive *in vivo* data, glycocalyx thickness on cultured endothelial cells has been found to be on the order of tens: By electron microscopy, Chappell et al. [14] found a glycocalyx thickness ranging from only 29 to 118 nm on primary HUVECs, whereas perfusion-fixed umbilical vein glycocalyx measured 878 nm. A similar discrepancy was found by Potter and Damiano [44], who detected a hydrodynamically relevant glycocalyx thickness of less than 30 nm on HUVECs cultivated in perfused microchannels. Based on these findings, it has been a matter of debate if this discrepancy is due to methodological limitations, or if culturing conditions impede the assembly of a mature eGC *in vitro* [45].

In contrast to those previous reports, our AFM data provides evidence for the existence of a mature, enzyme-sensitive endothelial glycoclayx on both, freshly isolated rat/mouse aorta preparations and cultured endothelial cells by a linear approximation over the first hundreds of nanometers of the force indentation curves that represent the eGC. In contrast to the epithelial surface layer (entropic brush model) [26,28], the eGC is neither a stiff nor a homogeneous structure due to various electrostatic and molecular interactions between its constituents. The average *nanomechanical* thickness of the glycocalyx was 250 - 350 nm on *ex vivo* aortic preparations from mice and rats, respectively. Although heterogeneity and limitations of *in vivo* visualization methods probably impede any direct comparison of glycocalyx dimensions, we noted that this value was compatible with many of the aforementioned reports [19,39,44]. A possible explanation for the unexpected abundance of the glycocalyx seen *in vitro* is that we examined glycocalyx dimensions directly (i.e. physically) without exposing cultured endothelial cells to potentially harmful fixation or staining procedures, thereby avoiding impairment of the fragile glycocalyx structure. Moreover, it is important to know that the thickness of the eGC *in vivo* is not homogeneous and varies even within a single vessel, probably due to disturbed laminar blood flow [46].

When different types of endothelial cells were compared *in vitro*, we found quite similar glycocalyx nanomechanics regardless of vessel diameter (microvascular vs. macrovascular), culture conditions (primary culture vs. immortalized cell lines), vascular bed (aorta vs. brain vs. lung vs. umbilical cord), or species (human vs. rodent vs. bovine endothelial cells). This finding is surprising, given that glycocalyx dimensions of the macrocirculation presumably exceed those of the microcirculation, at least *in vivo* [15,47,48]. However, only recently, Bai et al found a 300 nm to 1µm thick enzyme-sensitive glycocalyx on cultured HUVECs by confocal microscopy [49]. From a technical perspective it is possible that our AFM approach can detect thickness and stiffness only in more dense glycocalyx-layers close to the plasma membrane [7,39], thereby underestimating (cell type specific) heterogeneity of the looser outer regions of the eGC. Another explanation could be that the absence of fluid shear stress and/or reduced abundance of plasma proteins may result in partial collapse of the glycocalyx during the AFM procedure. In the future, we will try to perform nanoindentation measurements under flowing conditions with various albumin concentrations to address these aspects further.

Glycocalyx research has traditionally focused on thickness as a surrogate of structural integrity. Although highly intuitive in principle, this concept provides no conclusions concerning the mechanical properties of this highly elute and deformable layer. Any change of the glycocalyx composition, for example by enzymatic digestion, has the potential to affect the way by which loading forces are transmitted to the underlying membrane and cytoskeleton [32]. It is reasonable to assume that glycocalyx stiffness is a surrogate of both, hydration and packing density of the glycocalyx. Using heparinase as a degrading enzyme, we have previously shown that the eGC stiffness correlates strongly with the amount of stained heparan sulfate residues [31]. Interestingly, here we could show that enzymatic removal of any of its main constituents caused considerable softening of the glycocalyx, which exemplifies the importance of considering the synergetic interaction of all glycocalyx constituents as a whole. 

Glycocalyx stiffness in the current study was slightly higher than in previous nanoindentation experiments performed on split-open arteries obtained from a human umbilical cord [29]. This difference appears likely due to different settings and samples used in the experiments. Only recently, O'Callaghan [50] and Bai [49] used nanoindentation to determine the Young's modulus (=elastic stress) of the glycocalyx on bovine lung microvascular endothelial cells (0.26 kPa) and HUVECs (0.39 kPa), respectively. The mathematical model (Hertz contact theory) used to calculate the Young's modulus approximates that the indented material is isotropic, frictionless, non-conforming, and continuous. This assumption, however, does not account for the very complex nonlinear anisotropic multilayer structure of the eGC. Furthermore, the Hertz model provides different values for stiffness caused by various boundary conditions. Due to the variety of boundary conditions the Hertz contact theory is probably not adequate for describing nanomechanical properties of the eGC. Thus, the Young's modulus cannot be compared with stiffness, as derived by our simplified model of a linear fit. 

In sepsis, the glycocalyx has been generally understudied, in part because of the aberrancy of *in vitro* preparations and its putative degradation during tissue handling [51]. Experimental studies in rats [40], pigs [41], and humans [42] have shown that LPS administration induces a significant rise in circulating glycocalyx constituents, such as heparan sulfate and syndecan-1. In addition, TNF-α has been shown to induce glycocalyx shedding in a hamster cremaster [52] and isolated guinea pig heart model [4]. Only recently, a groundbreaking intra-vital microscopy study by Schmidt et al. [53] provided compelling evidence, that LPS-induced vascular hyperpermeability and leukocyte adhesion in murine lungs is initiated by TNF-α-dependent degradation of the pulmonary endothelial glycocalyx [53]. However, complex *in vivo* imaging studies usually preclude rapid and economic identification of underlying molecular mechanisms. Here we used a novel AFM-based protocol to analyze deterioration of the eGC in the context of experimental sepsis. In accordance with the above mentioned reports we could depict severe glycocalyx damage in response to LPS-treatment *in vivo*. Of note, *in vivo* effects of LPS were essentially reproducible on HPMECs *in vitro*, demonstrating that glycocalyx damage in response to LPS does not require additional (non-endothelial) effector cells. Nanomechanical changes induced by TNF-α and thrombin, respectively, were identical to those seen after LPS exposure, suggesting that glycocalyx deterioration is not confined to endotoxemia, but probably occurs across the whole spectrum of critical illness. Consistent with this notion, increased plasma levels of shed glycocalyx constituents have been reported in patients with major trauma [54], acute lung injury [53], and after major vascular surgery with global or regional ischemia [55,56].

Some limitations regarding our study need to be addressed. First of all the eGC slope of the force separation curve may partially contain simultaneous deformation of the plasma membrane and the underlying cortical actin web (cell cortex). At least heparinase I did not affect cortical stiffness of the cell *per se*. Of course it would be very interesting to analyze the eGC alone (i.e. without the cell cortex). However, we are not aware of an experimental setup which would enable us to address this aspect further. Second, in contrast to a sharp AFM tip, the spherical tip used in this study is incapable of penetrating the eGC, but it is able to compress this surface structure to a maximum extent. Due to the fact that the eGC is a well compressible structure, it is important to mention, that the AFM approach of this study gives an indication of the apparent thickness, rather than the true anatomical thickness of the eGC. Finally, we cannot exclude, that a possible change of the cortical stiffness in response to inflammatory mediators might have influence thickness and stiffness of the glycocalyx. However, preliminary experiments have shown that softening of the cell cortex does not change the measured thickness and stiffness of the eGC. 

## Conclusion

Our findings suggest that AFM might serve as a novel tool to investigate glycocalyx pathobiology in cultured endothelial cells. This approach not only allows the quantification of thickness but also opens up a mechanical dimension (stiffness) for the investigation of glycocalyx damage in experimental sepsis. Understanding deterioration and refurbishment of the eGC in the context of sepsis will be pivotal in order to develop therapeutic strategies against vascular leakage and eventually improve outcome critically-ill patients.

## Supporting Information

Figure S1 **Effect of heparinase on cortical stiffness.** Treatment with heparinase I (1.0 S-U/mL, 60 min) *in* *vitro* has no significant effect on the stiffness of the plasma membrane and the underlying cortical actin web (cell cortex) on freshly-isolated rat aorta preparations. Data shown are from independent AFM experiments using paired aorta preparations derived from two rat aortas (indicated by either green or blue dots). Each dot represents an average of ≥5 force indentation curves per tip-position (i.e. per individual endothelial cell). ns, not significant. Horizontal bars indicate mean values.
(EPS)

Figure S2 **Inhibition of heparinase by heparin *in vitro.*** The decrease of eGC thickness and stiffness after incubation with heparinase I for 60 min is completely abolished by addition of heparin, a natural substrate for heparinase I. Data shown are from independent AFM experiments using paired aorta preparations from three rat aortas (indicated by color/shape). Each dot represents an average of ≥5 force indentation curves per tip-position (i.e. per individual endothelial cell). *P<0.0001 versus untreated; #P<0.0001 vs. heparin; ‡*P*<0.0001 vs. heparin plus heparinase. Horizontal bars indicate mean values.
(EPS)

Figure S3 **Effect of enzyme mix on glycocalyx properties.** The decrease of eGC thickness and stiffness after incubation with the combination of heparinase, hyaluronidase and chondroitinase for 60 min reduced thickness and stiffness even more effectively than the individual enzymes. Data shown are from independent AFM experiments using paired aorta preparations (indicated by color/shape). Each dot represents an average of ≥5 force indentation curves per tip-position (i.e. per individual endothelial cell). *P<0.0001 versus buffer; #P<0.0001 vs. heparinase; ‡*P*<0.05 vs. chondroitinase, †*P*<0.001 vs. hyaluronidase. Horizontal bars indicate mean values.
(EPS)

Figure S4 **Comparison of rat and mouse aorta.** Enzymatic removal of heparan sulfate residues by heparinase I reduced eGC thickness (A) and stiffness (B) on aorta preparations from rats or mice, respectively. Data shown are from independent AFM experiments using paired aorta preparations from three aortas per species. Each bar represents an average of 5 force indentation curves per tip-position (i.e. per individual endothelial cell) and in summary five tip-positions per aorta patch (= 15 values per bar). *P<0.0001.
(EPS)
